# Dietary soyasaponin supplementation to pea protein concentrate reveals nutrigenomic interactions underlying enteropathy in Atlantic salmon (*Salmo salar*)

**DOI:** 10.1186/1746-6148-8-101

**Published:** 2012-07-02

**Authors:** Trond M Kortner, Stanko Skugor, Michael H Penn, Liv Torunn Mydland, Brankica Djordjevic, Marie Hillestad, Aleksei Krasnov, Åshild Krogdahl

**Affiliations:** 1 Aquaculture Protein Centre (a CoE), Department of Basic Sciences and Aquatic Medicine, Norwegian School of Veterinary Science, Oslo, Norway; 2 Nofima Marin, Ås, 1432, Norway; 3 Aquaculture Protein Centre (a CoE), Department of Animal & Aquacultural Sciences, Norwegian University of Life Sciences, Ås, N-1432, Norway; 4 Biomar AS, Nordre Gate 11, Trondheim, 7011, Norway

**Keywords:** Plant protein sources, Fish feed, Microarray, Inflammation, Digestion, Saponin

## Abstract

**Background:**

Use of plant ingredients in aquaculture feeds is impeded by high contents of antinutritional factors such as saponins, which may cause various pharmacological and biological effects. In this study, transcriptome changes were analyzed using a 21 k oligonucleotide microarray and qPCR in the distal intestine of Atlantic salmon fed diets based on five plant protein sources combined with soybean saponins.

**Results:**

Diets with corn gluten, sunflower, rapeseed or horsebean produced minor effects while the combination of saponins with pea protein concentrate caused enteritis and major transcriptome changes. Acute inflammation was characterised by up-regulation of cytokines, NFkB and TNFalpha related genes and regulators of T-cell function, while the IFN-axis was suppressed. Induction of lectins, complement, metalloproteinases and the respiratory burst complex parallelled a down-regulation of genes for free radical scavengers and iron binding proteins. Marked down-regulation of xenobiotic metabolism was also observed, possibly increasing vulnerability of the intestinal tissue. A hallmark of metabolic changes was dramatic down-regulation of lipid, bile and steroid metabolism. Impairment of digestion was further suggested by expression changes of nutrient transporters and regulators of water balance (e.g. aquaporin, guanylin). On the other hand, microarray profiling revealed activation of multiple mucosal defence processes. Annexin-1, with important anti-inflammatory and gastroprotective properties, was markedly up-regulated. Furthermore, augmented synthesis of polyamines needed for cellular proliferation (up-regulation of arginase and ornithine decarboxylase) and increased mucus production (down-regulation of glycan turnover and goblet cell hyperplasia) could participate in mucosal healing and restoration of normal tissue function.

**Conclusion:**

The current study promoted understanding of salmon intestinal pathology and establishment of a model for feed induced enteritis. Multiple gene expression profiling further characterised the inflammation and described the intestinal pathology at the molecular level.

**Ethical approval:**

The present experiment was approved by the Norwegian Animal Research Authority and conducted according to prevailing animal welfare regulations: FOR-1996-01-15-23 (Norway), European Convention for the Protection of Vertebrate Animals used for Experimental and Other Scientific Purposes (Strasbourg, 18.III.1986) and COUNCIL DIRECTIVE of 24 November 1986 on the approximation of laws, regulations and administrative provisions of the Member States regarding the protection of animals used for experimental and other scientific purposes (86/609/EEC).

## Background

In aquaculture, there is a growing demand for alternative plant-based feed ingredients to replace traditionally used fish meal [1]. However, most plant-derived nutrient sources contain various antinutritional factors (ANFs) such as saponins, which may exert harmful effects when ingested by animals [2,3]. Saponins are triterpenoidal or steroidal glycosides naturally occurring in many feed ingredients of plant origin such as soy, pea, sunflower and lupin. Various pharmacological and biological effects of saponins have been reported [4-6], and many of these have been attributed to the amphiphilic structure of saponins. Saponins can affect intestinal condition and functions in different ways. The ability of saponins to interact with sterols may account for many of the reported biological effects, particularly those that involve membrane properties. Saponins bind to membrane cholesterol and seem to increase cellular permeability, which may in turn have significant effects on the uptake of macromolecules including allergens [7] and antigens [4]. Based on their detergent and surfactant properties, dietary saponins likely disturb fat emulsification and formation of micelles and absorption of their constituents, i.e. bile salts, fatty acids, fat-soluble vitamins and other lipid soluble compounds. In mammals, saponins may decrease lipid and protein digestibility [4] as well as reduce absorption of iron [8] and fat-soluble vitamins A and E [9]. Most saponins can form complexes with intestinal bile salts and cholesterol [10], thus decreasing intestinal cholesterol reabsorption [4]. Another possible mode of saponin hypocholesterolemic action is through loss of cell membrane cholesterol from shed cells via increased intestinal cell turnover rate due to the membranolytic action of saponins [11].

Feeding salmonid fishes with diets containing high inclusion levels of soybean meal (SBM), a saponin rich ingredient, have in most experiments caused a dose dependent distal intestinal inflammation (enteritis) [12,13]. Recently, we demonstrated that high dietary levels of another potential alternative protein source, pea protein concentrate (PPC), induced inflammation in the distal intestine of Atlantic salmon similar to that described for SBM-induced enteritis [14]. Peas also contain high levels of saponins. The causative factor for the SBM- and PPC-induced enteritis in salmonids has not been conclusively identified, but there are strong indications that saponins are involved in the stimulation of cytokine production [15] and induction of inflammation [16,17]. However, pure saponins will not induce enteritis unless some other plant components are present [3].

The effects of various plant protein sources on fish growth performance, nutrient digestibility and gut health have been extensively studied [1]. In contrast, only fragmentary information on the impacts of plant-derived ANFs on fish health is currently available, and the molecular mechanisms remain unknown. In the present study, we addressed possible interactions between soyasaponins and five different plant protein sources. The five plant ingredients all have potential as alternative protein source in aquafeeds and were included at levels as high as practically possible in commercial diet formulations. Given the limited knowledge of saponin effects on fish, it is expedient to apply high-throughput analytic techniques. Consequently, multiple gene expression profiling with an oligonucleotide microarray was conducted to investigate the transcriptomic responses of Atlantic salmon distal intestine to dietary saponin at inclusion levels naturally present in soy. This work was part of a larger feeding trial, and fish performance and physiological data have been reported in detail elsewhere [18].

## Results

### Fish performance

Fish performance data are presented in detail elsewhere [18]. In brief, saponin inclusion significantly decreased feed intake and body weight for the PPC-based diet. For the other diets, feed intake and body weight seemed to be constant or slightly increased. Saponin supplementation showed no significant effects on the feed efficiency ratio.

### Histology

**Figure 1:**
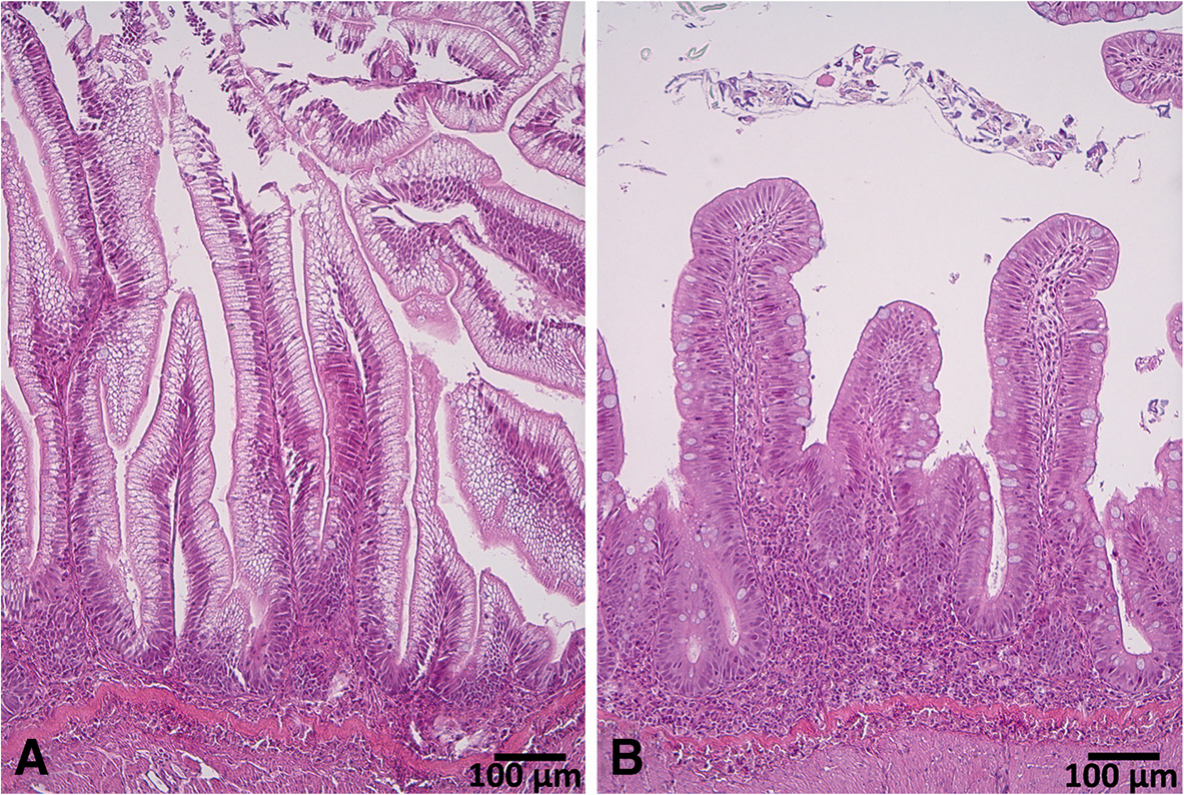
**Representative images of distal intestine tissue from fish fed diet PPC (A) and fish fed diet PPC + S (B).** The tissue from PPC + S fed fish showed clear signs of intestinal inflammation including shortened mucosal folds, fusion between adjacent folds and a prominent inflammatory infiltrate.

**Figure 2:**
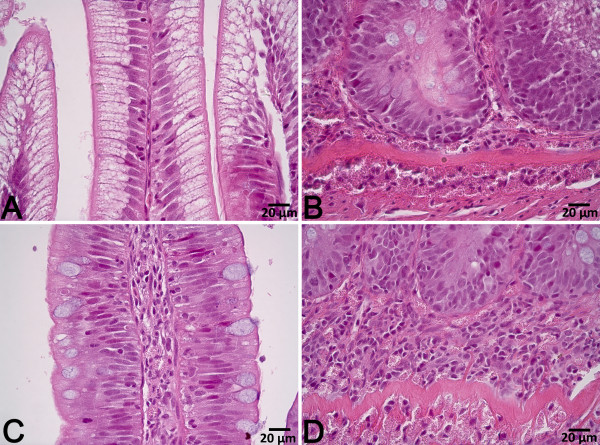
**Representative images of distal intestine tissue from fish fed diet PPC (A & B) and fish fed diet PPC + S (C & D).** The tissue from PPC + S fed fish exhibited reduced enterocyte vacuolization and abnormal nucleus position, increased lamina propria and submucosa width with prominent leukocyte infiltration.

Saponin supplementation markedly affected distal intestine histology when supplemented to the diet containing PPC (Figure 1 and 2). The changes observed in the PPC + S diet group included typical enteritic changes such as higher degrees of mucosal fold fusion (bridging), connective tissue hyperplasia and leukocyte infiltration in the lamina propria and submucosa, reduced supranuclear absorptive vacuolization and abnormal nucleus position in enterocytes, and increased numbers of goblet cells. Distal intestine histology was either minimally, or not, affected in all other diet groups. Slightly shorter mucosal folds and a wider lamina propria were observed in fish fed rapeseed meal (RSM), and higher numbers of goblet cells were observed in fish fed sunflower meal (SFM). However, no clear signs of inflammation were present. Quantitative histology results are presented in detail elsewhere [18].

### Transcriptomic responses: An overview

**Figure 3:**
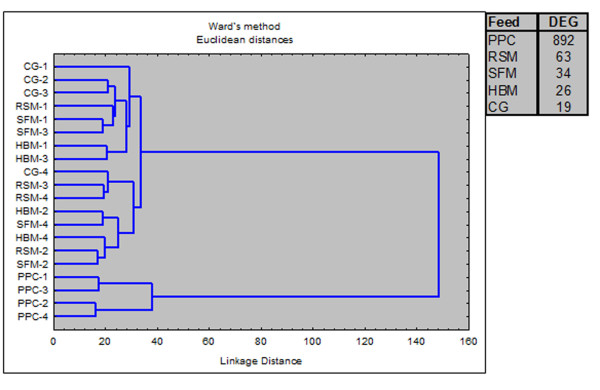
**Clustering of microarray samples and numbers of differentially expressed genes (DEG) after saponin supplementation to five plant protein sources.** Abbreviations: CG, corn gluten; PPC, pea protein concentrate; SFM, sunflower meal; RSM, rapeseed meal and HBM, horse bean meal.

**Figure 4:**
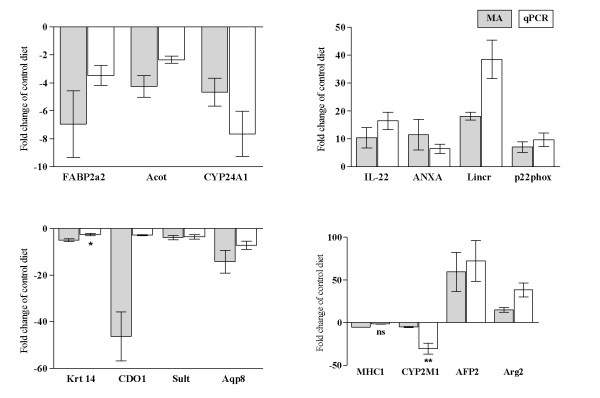
**Comparison of qPCR and microarray (MA) results.** Data are presented as fold changes of PCC control diet group. All MA results (n = 4 fish/group) are significant. For qPCR results (n = 9 fish/group), data differences between PPC and PPC + S group are denoted as * = p < 0.05, ** = p < 0.01 and ns = not significant. For genes not denoted, p < 0.0001. See Table 2 for acronym explanations.

A sizeable effect of saponins (S) on the distal intestinal transcriptome was observed only in combination with pea protein concentrate (PPC). The number of differentially expressed genes (DEG) in the PPC + S diet group was 892, much higher than the other groups which ranged between 19 and 63 DEG. Hierarchical clustering separated PPC while other samples were joined in one cluster (Figure 3). As saponin supplementation to diets with corn gluten (CG), SFM, RSM and horsebean meal (HBM) did not produce any adverse effects on the intestine, further presentation focuses only on the effects of saponins in combination with PPC. A search for enriched terms in lists of DEG was applied for rapid screening of the thematic associations of the transcriptomic responses (Table 1). Results suggested that the PPC + S diet induced inflammation mediated by chemokines and complement components. The metabolic changes involved a number of pathways of amino acid, steroid and lipid metabolism. Effects on glutathione and xenobiotic metabolism could impair protection against reactive oxygen species (ROS) and toxicity, while protein folding was a hallmark of cellular stress. Effects of PPC + S diet on higher levels of biological organization were observed by terms related to cellular and tissue structures (cell surface, lysosome, mitochondrion, peroxisome and basal membrane) and integrative functions (hormone activity, digestion). For validation of microarray results with qPCR, 15 genes related to the key functional groups were selected (Table 2) and the data produced with two independent methods were closely correlated (Pearson's correlation coefficient: 0.80, *p* = 0.0004). Data are presented in Figure 4.

**Table 1:** Functional GO categories and KEGG pathways enriched with genes that were differentially expressed in response to a combination of PPC and saponins

**GO categories and KEGG pathways**	Features^*^	**p-value**^**†**^
Inflammatory response	26/236	<0.001
Complement and coagulation cascade	10/92	0.026
Antigen processing and presentation	9/77	0.022
Chemokine activity	5/6	<0.001
Valine, leucine and isoleucine degradation	17/79	<0.001
Arginine and proline metabolism	14/107	<0.001
Tryptophan metabolism	12/60	<0.001
Tyrosine metabolism	10/51	<0.001
Glycine, serine and threonine metabolism	10/56	<0.001
Lysine degradation	8/57	0.009
Beta-alanine metabolism	6/41	0.022
Lipid metabolic process	27/300	0.004
Retinol metabolism	18/79	<0.001
Fatty acid metabolic process	14/97	<0.001
Glycerolipid metabolism	13/68	<0.001
C21-steroid hormone metabolism	8/49	0.002
Sphingolipid metabolism	7/51	0.018
Carbohydrate metabolic process	22/264	0.023
Mitochondrion	78/1091	0.002
Lysosome	20/183	<0.001
Protein folding	19/180	0.002
Peroxisome	16/118	<0.001
Cell surface	15/136	0.004
Metabolism of xenobiotics	12/55	<0.001
Glutathione metabolism	12/69	<0.001
Glycosaminoglycan degradation	5/32	0.031
Extracellular space	28/367	0.032
Basement membrane	9/68	0.008
Hormone activity	8/41	<0.001
Digestion	7/49	0.014

^*^ Numbers of genes among DEG and on the microarray platform.

^†^ Yates’ corrected chi-square.

**Table 2:** Primers used in qPCR assays

**Genbank acc.**	**Gene**	**Primer sequence (5’-3’)**	**Function**	**Amplicon (bp)**	**Primer efficiency**
**BU694011**	Keratin 14 (Krt14)	F: CAAGGTGGTGATCGTCACAG R: TGGGACCTTAAGAAGCGTGT	Cytoskeleton, cell shape	83	1.91
**DW572073**	Interleukin 22 (IL22)	F: GGAGAAGCAGGACAAGCATC R: ATAGCACAGCCGTGTTCCTT	Immune	93	1.89
**209733777**	MHC class I (MHCI)	F: CTCAGTCACGCAAGAGCAAG R: AGCCATGTTTCCACTGAAGG	Immune: antigen presentation	111	2.04
**BT047112**	E3 ubiquitin-protein ligase LINCR (Lincr)	F: CTGGGGACACCTTCAGACAT R: TACGCATAGCTCCACACCAG	Immune: effector	114	1.89
**DY692748**	Type-2 ice-structuring protein (AFP2)	F: GGTTGCAGCAGCACCTAAA R: CCGAGGAGTGTTCACAAACAT	Immune: lectin	100	1.90
**EG833741**	Cytochrome b558 alpha-subunit (p22phox)	F: GGCACCAGCGTAGAAAGAAC R: GCAGATCGCTGCATGTAGAA	Immune: oxidative burst	111	2.02
**223647763**	Arginase-2, mitochondrial (Arg2)	F: GACAGGCTCGGCATTCAGA R: AAAGACGGGTCCATCGCAT	Immune, amino acid metabolism	110	2.05
**CA060324**	Annexin A1 (ANXA)	F: GTCAGAATCTTGGTCCTGGTTC R: ACTGCCGTAGTGAAGTGTGCT	Inflammation, exocytosis	98	2.04
**DW531828**	Cysteine dioxygenase type 1 (CDO1)	F: TCATTGCTCTCGCTCTGCT R: GAGTTATTGCCAATGAGCTTCAG	Metabolism: amino acids	82	2.00
**DY730337**	Sulfate transporter (Sult)	F: AGGCAAAAGAGATCCCAGGT R: CCCAATGTCAATACCGCTCT	Metabolism: bile, glutathione, xenobiotics	113	2.01
**CA041487**	Fatty acyl-CoA hydrolase, medium chain (Acot)	F: GGTCCCTCTTCAGGTGTTGA R: TTTGCTCGTACAGGGTCTCC	Metabolism: fatty acids	121	1.97
**DW533963**	Fatty acid-binding protein, intestinal (FABP2a2)	F: CAGCTACGATGGAGTCGAAGCCA R: GGTTGTAAAATGTTCAGTGTCAC	Metabolism: fatty acids	139	1.96
**EG934966**	Cytochrome P450 24A1 (CYP24A1)	F: GCGTGTTACCCAGGATGAGT R: GGGAAATTCTCCTCGTCCAT	Metabolism: steroids	105	1.96
**DY728977**	Cytochrome P450 2 M1 (CYP2M1)	F: TCAGTCCCACCTCTGTACCC R: AATTTGGGATCAGCAAGCA	Metabolism: xenobiotic	118	1.97
**DW532464**	Aquaporin-8 (Aqp8)	F: GTTGGCATAGTTCTCCTTTGATG R: TTTCAACCCTCCCTTCACC	Water channel	148	1.96
**AF321836**	Elongation factor 1A (EF1A)	F: GTGCTGTGCTTATCGTTGCT R: GGCTCTGTGGAGTCCATCTT	Translation factor	148	1.91
**BT050045**	Glyceraldehyde-3-phosphate dehydrogenase (GAPDH)	F: AAGTGAAGCAGGAGGGTGGAA R: CAGCCTCACCCCATTTGATG	Glycolytic enzyme	96	1.89
**BT043501**	Hypoxanthine phospho-ribosyltransferase 1(HPRTI)	F: CCGCCTCAAGAGCTACTGTAAT R: GTCTGGAACCTCAAACCCTATG	Purine metabolism	255	1.99
**BG936649**	RNA polymerase II (RNAPOLII)	F:CCAATACATGACCAAATATGAAAGG R: ATGATGATGGGGATCTTCCTGC	DNA transcription	157	1.85

### Inflammatory responses

Salmon fed PPC + S showed increased distal intestinal transcription of genes involved in inflammation at different levels (Table 3). Up-regulation was observed in several chemokines, cytokines, especially IL-22 (up-regulated 10-fold), as well as chemokine and cytokine receptors. Two genes for proteins of eicosanoid metabolism were induced 2- to 3-fold (arachidonate 5-lipoxygenase-activating protein, leukotriene b4 12-hydroxydehydrogenase). Annexins contribute to the intestinal resistance to injury, as they possess anti-inflammatory properties as well as gastroprotective properties. Microarray analyses did not show increase of IL-1 and TNFalpha. However, up-regulation of NFkB, several functionally related pro-inflammatory transcription factors and TNF-induced proteins was revealed and this suggested an acute character of inflammation. Interestingly, this was in parallel with the down-regulation of MHCI (down 4.3-fold) and several virus responsive genes that are dependent on IFN. Diverse immune effector mechanisms were activated. Up-regulation was shown for lectins and genes with reported induction in pathogen infected fish. Stimulation of different complement pathways was likely since 2- to 4-fold up-regulated levels were shown for genes associated with classical (C1Q-like proteins and IG receptors) and alternative (factors P and D) pathways. This was in parallel with decreased expression of negative complement regulators: C1 inhibitor (5-fold down-regulated) and C4b-binding protein (1.8-fold down-regulated). Activation was shown by several extracellular matrix (ECM) degrading proteases including matrix metalloproteinases (MMPs) and their inhibitors that are commonly co-regulated. Inflammatory tissue damages were also suggested by up-regulation of several components of the respiratory burst complex that generates ROS, i.e. cytochromes b-245 and b558 (up-regulated 3- and 7-fold, respectively), myeloperoxidase (up 32-fold) and neutrophil cytosolic factor 1 (up 7-fold). Arginase 2 (ARG2, up 15-fold) and ornithine decarboxylase (ODC, up 1.8-fold) can direct the arginine flux away from nitric oxide synthase and nitrogen nitric oxide (NO) production, a free radical toxic to bacteria but also an important signalling molecule. The oxidative stress was probably reinforced by down-regulation of diverse ROS scavengers and proteins that bind iron and heme; both are potent catalysts of free radicals production.

**Table 3:** **Differentially expressed genes in PPC + S group involved in immune and inflammatory responses** (**mean fold change of PPC control group levels)**

***Gene***	***Fold change ± SE***
***Inflammatory mediators and transducers***	
Interleukin-22	10.35 ± 3.67
Interleukin-18	2.66 ± 0.84
Chemokine CK-1	4.75 ± 0.99
C-C motif chemokine 19-1	3.73 ± 1.12
Suppressor of cytokine signaling 1	2.68 ± 0.33
C-C chemokine receptor type 9	2.57 ± 0.68
C-C motif chemokine 21	−1.95 ± 0.30
Interleukin-6 receptor subunit alpha	2.73 ± 0.98
Interleukin-1 receptor type II	2.07 ± 0.30
Interleukin-1 receptor antagonist	1.65 ± 0.09
Arachidonate 5-lipoxygenase-activating protein	3.49 ± 0.91
Leukotriene b4 12-hydroxydehydrogenase	2.43 ± 0.40
Annexin A1	11.46 ± 5.46
Annexin A2-A	2.13 ± 0.20
Annexin A5	2.39 ± 0.33
TNF decoy receptor	5.37 ± 1.26
TNF receptor superfamily member 5	1.83 ± 0.21
TNFalpha-induced protein 8-like protein 2	1.78 ± 0.21
NF-kappa-B p100 subunit	2.14 ± 0.07
NF-kappa-B inhibitor alpha	1.71 ± 0.03
NF-kappa-B inhibitor epsilon	1.96 ± 0.14
CCAAT/enhancer binding protein beta-2	3.00 ± 0.98
Transcription factor AP-1	1.67 ± 0.20
Transcription factor jun-B	2.16 ± 0.32
***IFN-dependent***	
MHC class I antigen	−4.27 ± 0.95
MHC class I	−5.42 ± 0.16
MHC class Ia heavy chain	−3.76 ± 1.56
Beta-2 microglobulin	−2.27 ± 0.52
Tyrosine-protein kinase Jak1	2.14 ± 0.26
Similar to very large inducible GTPase 1	−5.81 ± 1.54
Receptor-transporting protein 3	−3.52 ± 0.91
Gamma-interferon-inducible thiol reductase	−2.67 ± 0.65
Interferon-induced protein 44	−2.48 ± 0.39
Fish virus induced TRIM protein	−2.12 ± 0.37
SRK2 tyrosine kinase	−1.89 ± 0.24
Galectin-3-binding protein	−1.74 ± 0.22
***Effectors: complement and lectins, antimicrobial proteins***	
FBPL4 (lectin)	7.46 ± 2.29
Precerebellin-like protein (lectin)	8.58 ± 4.27
Complement factor D	4.46 ± 1.93
Complement C1q-like protein 2	3.34 ± 1.41
Complement C1q-like protein 4	2.26 ± 0.59
C5a anaphylatoxin chemotactic receptor	2.34 ± 0.61
Complement component C6	2.45 ± 0.65
Properdin P factor 2	1.76 ± 0.17
C1 inhibitor	−5.25 ± 1.31
C4b-binding protein alpha chain	−1.77 ± 0.16
Nattectin	5.97 ± 2.21
Cathelicidin	7.08 ± 3.32
High affinity IG epsilon receptor gamma	3.32 ± 1.16
High affinity IG gamma Fc receptor I	1.85 ± 0.11
Differentially regulated trout protein 1	3.39 ± 1.28
C type lectin receptor A	2.20 ± 0.48
***Proteases and inhibitors, T-cells***	
E3 ubiquitin-protein ligase LINCR	18.11 ± 1.38
Matrix metalloproteinase-9	3.56 ± 1.34
Collagenase 3	4.38 ± 1.74
Matrix metalloproteinase	2.97 ± 1.03
E74-like factor 3	2.78 ± 0.31
Metalloproteinase inhibitor 2	4.67 ± 1.44
Leukocyte elastase inhibitor	1.75 ± 0.06
T-cell receptor beta chain T17T-22	6.73 ± 1.91
T-cell immunoglobulin and mucin domain-containing 4	2.30 ± 0.51
CD86	1.90 ± 0.24
CTLA4-like protein	2.04 ± 0.22
***Oxidative burst, protection against free radicals***	
Myeloperoxidase	32.43 ± 4.77
Cytochrome b558 alpha-subunit	7.06 ± 1.86
Cytochrome b-245, beta polypeptide	3.08 ± 1.12
Neutrophil cytosolic factor 1	7.19 ± 3.88
Arginase-2, mitochondrial	14.78 ± 2.87
Ornithine decarboxylase 1	1.76 ± 0.26
Nitric oxide synthase trafficker	−1.67 ± 0.25
Glutathione reductase, mitochondrial	2.30 ± 0.34
Glutathione peroxidase 4b	−2.05 ± 0.12
Glutathione S-transferase alpha 3	−2.63 ± 0.36
Glutathione S-transferase kappa 1-like	−1.88 ± 0.25
Glutathione S-transferase P	−2.72 ± 0.67
Glutathione transferase zeta 1 isoform 1	−1.96 ± 0.12
Peroxiredoxin-4	−1.69 ± 0.14
Catalase	−2.47 ± 0.05
Arrestin domain-containing protein 2	−2.59 ± 0.13
CDGSH iron sulfur domain-containing protein 1	−2.50 ± 0.32
Peptide methionine sulfoxide reductase	−1.77 ± 0.21
5-aminolevulinate synthase, nonspecific, mitochondrial	3.54 ± 0.46
Metallothionein A	−6.46 ± 0.95
Heme oxygenase	−6.58 ± 1.78
Heme-binding protein 2	−2.89 ± 0.44
Ferritin, middle subunit	−3.03 ± 0.26
High affinity copper uptake protein 1	−2.62 ± 0.52
Copper transport protein ATOX1	−3.41 ± 0.80

### Metabolism

Unlike polyfunctional arginase 2, a suite of genes involved in several pathways of amino acids metabolism were down-regulated (Table 4). Reduction of amino acid- /peptide absorption could be predicted from down-regulation of B0,+ − type amino acid transporter (down 3.6-fold), sodium-dependent neutral amino acid transporter B0 (down 14-fold), taurine transporter and solute carrier family 15 member 1 (peptide transporter, down 4-fold). Decreased expression was found in a number of transporters that are known to or can take part in intestinal absorption of diverse nutrients including phosphates, copper and sulphate, sugars (facilitated and sodium-dependent transporters), lactate and pyruvate (monocarboxylate transporter 9), citrate (solute carrier family 13 member 3), other organic cations (solute carrier family 22 member 7) and vitamins (transcobalamin). Water channels (aquaporins) were down-regulated (Aqp8 showed a 14-fold reduction) while increased expression was shown by three chloride transporting proteins.

**Table 4:** **Differentially expressed genes in PPC + S group related to metabolism** (**mean fold change of PPC control group levels)**

***Gene***	***Fold change ±SE***
***Amino acid metabolism***	
Cysteine dioxygenase type 1	−46.28±10.52
L-pipecolic acid oxidase	−10.32±1.68
D-amino-acid oxidase	−2.95±0.66
Kynurenine 3-monooxygenase	−3.39±0.27
Gamma-glutamyltransferase 5	−2.84±0.63
Aspartate aminotransferase, cytoplasmic	−4.03±0.99
Aspartate aminotransferase, mitochondrial	−1.85±0.18
4-aminobutyrate aminotransferase	−3.85±1.04
Alpha-aminoadipate aminotransferase	−2.13±0.32
Guanidinoacetate N-methyltransferase	−7.45±1.38
Arginine N-methyltransferase 3	2.48±0.42
Diamine acetyltransferase 2	−2.65±0.70
Amidohydrolase domain containing 1	−3.01±0.84
Gamma-glutamyl hydrolase	−1.91±0.22
Methylmalonate-semialdehyde dehydrogenase	−3.85±0.85
3-hydroxyisobutyrate dehydrogenase	−1.80±0.12
Methionine-R-sulfoxide reductase B2	−2.60±0.67
Lysine ketoglutarate reductase	−2.29±0.51
Glycine cleavage system H protein	−2.29±0.53
Glutaminase	−3.56±0.31
Adenylosuccinate synthase like 2	−1.74±0.05
Folylpolyglutamate synthase	−2.14±0.22
Ammonium transporter Rh type B	−3.99±1.73
***Transporters***	
B0,+−type amino acid transporter 1	−3.60±1.00
Sodium-dependent neutral amino acid transporter B0	−14.12±7.68
Taurine transporter	−1.85±0.27
Sodium-dependent phosphate transport protein 2A	−6.13±2.69
Facilitated glucose transporter, member 8	−4.08±1.68
Facilitated glucose transporter member, 11	−1.9±0.40
Solute carrier family 15 member 1	−4.11±0.98
Sodium/glucose cotransporter 2	−2.15±0.47
Monocarboxylate transporter 9	−3.31±0.81
Solute carrier family 22 member 7	−4.35±1.16
ATPase, Cu++ transporting, beta	−2.25±0.44
Sulfate transporter	−3.86±0.94
Solute carrier family 13 member 3	−6.15±3.13
Two pore calcium channel protein 1	−3.77±1.03
Transcobalamin-2	−4.36±1.37
Aquaporin-8	−14.24±4.90
Aquaporin FA-CHIP	−1.89±0.28
Calcium activated chloride channel 2	1.83±0.33
Solute carrier family 12, member 8	2.53±0.49
Chloride channel protein 3	2.92±0.82
Sodium-coupled neutral amino acid transporter 2	2.32±0.45
Multidrug resistance associated protein 2	2.59±0.86
***Lipid and steroid metabolism***	
Peroxisome proliferator-activated receptor gamma	−1.98±0.30
StAR-related lipid transfer protein 5	1.93±0.33
25-hydroxycholesterol 7-alpha-hydroxylase	−1.94±0.13
Alpha-methylacyl-CoA racemase	−2.58±0.83
Sterolin 1	−3.14±0.92
Very long-chain acyl-CoA synthetase	−2.45±0.80
Fatty acyl-CoA hydrolase , medium chain	−4.25±0.78
Acyl-CoA dehydrogenase	−2.50±0.65
Isovaleryl-CoA dehydrogenase, mitochondrial	−2.12±0.42
Peroxisomal bifunctional enzyme	−2.21±0.51
Peroxisomal 3,2-trans-enoyl-CoA isomerase	−2.20±0.42
Hydroxyacid oxidase 2	−6.13±2.15
Fatty aldehyde dehydrogenase	−1.98±0.34
Acyl-CoA desaturase	−2.74±0.26
Delta-6 fatty acyl desaturase	−2.03±0.15
Peroxisomal trans-2-enoyl-CoA reductase	−1.93±0.26
Fatty acid desaturase domain family, member 6	−3.04±0.62
Acyl-coenzyme A thioesterase 5	−2.30±0.39
Ganglioside GM2 activator	−2.68±0.37
Dihydroxyacetone kinase 2	−2.06±0.25
1-acyl-sn-glycerol-3-phosphate acyltransferase delta	−2.06±0.32
Phospholipase B1	−3.81±1.22
Retinol dehydrogenase 8	−3.27±0.88
Lecithin retinol acyltransferase	−1.88±0.11
Beta-carotene 15, 15-monooxygenase 1	−4.8±1.20
Epidermal retinal dehydrogenase 2	−5.42±1.83
Dehydrogenase/reductase SDR family member 12	−2.19±0.31
Sphingomyelin phosphodiesterase 1, acid lysosomal 1	−2.62±0.61
3-oxo-5-beta-steroid 4-dehydrogenase	−2.25±0.57
Lipocalin	−1.67±0.09
Apolipoprotein A-I-1	−4.96±1.73
Apolipoprotein A-I-2	−4.74±1.75
Fatty acid-binding protein, intestinal	−6.95±2.39
Fatty acid-binding protein, liver-type	−2.23±0.29
apolipoprotein B	−2.91±0.06
Apolipoprotein A-IV	−1.90±0.35
Apolipoprotein A-II	−5.56±0.95
Carnitine palmitoyl transferase I	−1.86±0.17
Arylacetamide deacetylase	−3.56±0.31
2-acylglycerol O-acyltransferase 2-A	−3.89±1.40
N-acylsphingosine amidohydrolase 2	−6.64±2.12
***Proteases***	
Angiotensin I converting enzyme 2	−3.42±0.87
Aspartyl aminopeptidase	−1.97±0.32
Carboxypeptidase N catalytic chain	−4.06±0.90
Cathepsin K	1.86±0.22
Cathepsin L.1	−7.30±2.89
Cathepsin M	−1.82±0.14
Glutamyl aminopeptidase	−1.86±0.26
Legumain	−4.17±1.25
Meprin A, alpha	−4.10±1.38
Metalloproteinase inhibitor 3	−12.75±5.59
Pancreatic secretory trypsin inhibitor	−2.35±0.21
Peptidase D	−2.99±0.79
Probable serine carboxypeptidase CPVL	−4.08±1.21
Serine carboxypeptidase 1	−3.99±1.30
Xaa-Pro aminopeptidase 1	−2.09±0.45
N-acetylated alpha-linked acidic dipeptidase-like 1	−2.55±0.6
Digestive cysteine proteinase 2	−3.37±0.94
Lactase-phlorizin hydrolase preproprotein	−8.55±2.13
***Xenobiotic metabolism***	
Alanine--glyoxylate aminotransferase 2	−1.91±0.17
Aldehyde dehydrogenase family 9 member A1-A	−1.82±0.14
Succinate-semialdehyde dehydrogenase, mitochondrial	−2.40±0.36
Aryl hydrocarbon receptor nuclear translocator-like	−2.28±0.6
Arylamine N-acetyltransferase	−4.88±2.36
Cytochrome P450	−3.00±0.93
Cytochrome P450 24A1, mitochondrial	−4.67±1.00
Cytochrome P450 2M1	−5.15±0.55
Cytochrome P450 3A27	−2.44±0.57
Cytochrome P450 4F3	−2.47±0.47
Cytochrome P450 monooxygenase CYP2K1v2	−5.94±2.24
cytochrome P450, family 26, subfamily A1-2	−2.76±0.68
Epoxide hydrolase 2	−2.17±0.53
Fatty acid amide hydrolase 2	−2.05±0.13
Nitrilase homolog 2	−2.24±0.22
Probable thiopurine S-methyltransferase	−2.56±0.54
Aldehyde dehydrogenase, mitochondrial	−2.08±0.34
Sulfotransferase 6B1	−2.83±0.92
UDP-glucuronosyltransferase 2A2	−3.63±0.83

Slight induction of putative bile salt transporter (multidrug resistance associated protein, up 2.6-fold) was in parallel with down-regulation of genes for carriers of lipids including several apolipoproteins (2- to 5-fold down-regulated) and sterolin (cholesterol), steroids and retinoids (lipocalin), triglycerides and fatty acids. A 3.9-fold down-regulation was observed for 2-acylglycerol O-acyltransferase 2-A, which takes part in absorption of dietary fat in the small intestine by catalyzing the resynthesis of triacylglycerol in enterocytes. A number of down-regulated genes is involved in different pathways including production of bile (25-hydroxycholesterol 7-alpha-hydroxylase, alpha-methylacyl-CoA racemase), steroid metabolism, glycerolipids (dihydroxyacetone kinase, 1-acyl-sn-glycerol-3-phosphate acyltransferase delta), phospholipids (phospholipase B1) and retinol. Several down-regulated genes were implicated in different aspects of fatty acids utilization. Metabolism of lipids and conversion of toxic compounds include common steps since biotransformation of xenobiotics requires improvement of their solubility. This is achieved by processes grouped in phase I (introduction of polar groups) and phase II (attachment of small hydrophilic molecules). The PPC + S diet suppressed genes with important roles in detoxification. CYP450 are enzymes with different substrate specificity that play an essential part in phase I as well as several other enzymes presented in Table 4. Phase II reactions are catalyzed by glutathione S-transferases (Table 3), sulfotransferases and UDP-glucuronosyltransferases. The precise role of alanine-glyoxylate aminotransferase (down 1.9-fold) is not known, however this gene showed responses to various aquatic contaminants and was suggested as a marker of generalized toxicity [19]. Down-regulation of lysosomal proteases (cathepsins) could be attributed to the inflammatory response as well as digestion. Reduction of digestive proteolytic action could be predicted from down-regulation of several aminopeptidases and carboxypeptidases, as well as pancreatic secretory trypsin inhibitor, which protects against premature activity of trypsin.

### Tissue damage and repair

Up-regulation of several genes involved in exocytosis including annexins (Tables 3 and 5) was in accordance with increased production of mucus as indicated by increased numbers of goblet cells. Down-regulation was observed in several genes involved in epithelial cell-to-cell contacts including genes that encode tight junctions (TJs). The 2.5-fold down-regulation of the gap junction Cx32.2 could affect passage of inorganic ions and small water-soluble molecules in between cells. Suppression of the two cadherins (epithelial cadherin precursor and protocadherin 20, 3.1- and 2.2-fold down-regulated, respectively) is also worth mentioning as cadherin-mediated cell-cell and cell-matrix junctions have crucial roles both within normal and injured intestinal epithelium [20]. Surface glycans are essential for cell recognition and interactions. Three glycan modifying esterases were up-regulated (glucosamine 6-phosphate N-acetyltransferase, beta-1,3-N-acetylglucosaminyltransferase 7 (B3GN7) and alpha-1,3-fucosyltransferase), while several genes required for degradation and turnover of glycans were down-regulated, e.g. aspartylglucosaminidase (AGA, down 2.2-fold). Decreased expression was observed in growth factors and transcription regulators that control differentiation of various cell lineages including mesenchymal (class B basic helix-loop-helix proteins 2 and 3, down-regulated 1.7- and 5.9-fold, respectively) and endothelial (angiogenin and angiopoietin-related protein 4), fibroblasts and other elements of connective tissue. Furthermore, lactase-phlorizin hydrolase (LPH, 8.5-fold decrease) is exclusively expressed in the small intestine in mammals, and is often used as a marker for the differentiation of enterocytes, as LPH mRNA can first be detected in the transition zone between the crypt and the villus [21]. An interesting finding was the 2.4-fold up-regulation of putative guanylin, an intestinal peptide with an important role in regulation of water balance.

**Table 5:** **Differential expressed genes in PPC + S group involved in tissue homeostasis and integrative intestine functions** (**mean fold change of PPC control group levels)**

***Gene***	***Fold change ± SE***
***Exocytosis***	
Exosome complex exonuclease RRP42	1.85 ± 0.12
Exosome component Rrp46	1.75 ± 0.12
Gelsolin	9.28 ± 3.07
Signal recognition particle receptor subunit beta	2.47 ± 0.55
***Adhesion and glycans***	
Occludin	1.72 ± 0.21
Phospholipase D2	2.03 ± 0.18
Gap junction Cx32.2 protein	−2.54 ± 0.40
Rho-related GTP-binding protein RhoG precursor	1.79 ± 0.34
Kalirin, RhoGEF kinase isoform 3	1.63 ± 0.02
Myosin phosphatase-Rho interacting protein isoform 1	−1.88 ± 0.61
Myosin IB	15.3 ± 4.29
Tropomyosin alpha-3 chain	1.71 ± 0.24
Tropomyosin-1 alpha chain	1.77 ± 0.24
Protocadherin 20	−2.21 ± 0.29
Similar to laminin beta 2-like chain	−2.43 ± 0.51
Epithelial cadherin	−3.06 ± 0.61
Aspartylglucosaminidase	−2.23 ± 0.42
Glucosamine 6-phosphate N-acetyltransferase	1.92 ± 0.33
Beta-1,3-N-acetylglucosaminyltransferase 7	4.28 ± 0.68
Alpha-1,3-fucosyltransferase	2.65 ± 0.83
D-glucosaminylasparagine amidase F	−1.85 ± 0.08
Alpha-N-acetylgalactosaminidase	−2.29 ± 0.5
Glypican 1	−2.34 ± 0.39
Hyaluronan and proteoglycan link protein 4	−2.41 ± 0.16
Di-N-acetylchitobiase	−2.46 ± 0.56
N-acetylglucosamine-6-sulfatase	−3.16 ± 0.79
Beta-hexosaminidase beta chain	−3.45 ± 1.09
N-acetylneuraminate lyase	−4.3 ± 1.47
Mannosidase, alpha, class 2B, member 1	−8.25 ± 3.7
***Growth factors, regulators***	
Angiogenin-1	−2.11 ± 0.16
Bone morphogenetic protein 7	−2.11 ± 0.16
Angiopoietin-related protein 4	−2.31 ± 0.38
Connective tissue glrowth factor	−2.26 ± 0.32
Class B basic helix-loop-helix protein 2	−1.68 ± 0.19
Class B basic helix-loop-helix protein 3	−5.85 ± 2.34
Transmembrane glycoprotein NMB	−2.27 ± 0.10
TGFbeta-inducible early growth response protein 3	−2.38 ± 0.62
Guanylin	2.37 ± 0.34
Fibroblast growth factor 12	−2.53 ± 0.33
***Cellular stress***	
Ubiquitin	10.80 ± 2.85
60 kDa heat shock protein, mitochondrial	2.27 ± 0.54
Heat shock cognate 70 kDa protein	3.14 ± 0.31
Heat shock cognate 71 kDa protein	3.02 ± 0.26
Heat shock protein HSP 90-alpha	1.80 ± 0.09
Heat shock protein 67B2	−2.31 ± 0.38

## Discussion

In the present study, saponins negatively affected the intestine and caused lower growth performance, but only in combination with PPC. Histology of the distal intestine revealed enteritis resembling the changes associated with soy enteropathy [12,13], which was also confirmed with pronounced transcriptome changes. Moreover, microarray results were consistent with our previous report on soya induced gene expression changes in the distal intestine of Atlantic salmon [22]. Similar effects of SBM on expression levels of genes related to lipid, iron and xenobiotic metabolism have also been observed in the distal intestine of Atlantic halibut [23]. Interestingly, inflammation was not induced in the latter case, suggesting that suppression of some metabolic processes observed in the present work could occur independent of the activation of immune responses.

In previous studies, feeding 18% field peas [24] or 20% PPC [25] did not produce histomorphological changes, while high dietary PPC levels (35%) produced enteritis and adverse effects on growth performance and nutrient digestibility [14]. Peas have been reported to contain between 0.7 and 2.5 g kg^−1^ saponin [26-28]. However, the PPC used in the current work, produced by air classification, may have contained higher levels compared to unprocessed peas due to changes during processing [29-32]. The difference in distal intestinal morphology in fish fed the PPC + S diet could therefore be due to a dosage effect of saponins as suggested previously [16]. None of the other protein sources were expected to contain appreciable amounts of saponins [6,33-36]. Therefore, the basal saponin level in the different diets could explain the differences in the results. However, the level of saponin supplementation was equivalent to the amount found in a diet containing approximately 40% SBM, which consistently causes distal intestine inflammation. Moreover, similar histology was reported in fish fed either a fishmeal based control diet or the same diet supplemented with 2.6 g kg^−1^ of a 65% soyasaponin concentrate [16], an amount equivalent to a diet containing approximately 30% defatted SBM, a level which also consistently produces distal intestine inflammation. This indicates that some other component or contributing factor(s) is (are) necessary to induce an inflammatory response. The necessary factor may involve interactions with other ANFs present in peas, such as protease inhibitors, phytic acid, oligosaccharides, lectins, tannins, and/or dietary antigens [37,38], or changes in the intestinal microbiota [39].

The inflammatory response was characterized by marked involvement of genes regulating T-cell functions, in line with the high T-cell reactivity seen during the development of soy enteropathy [40]. Increase in the expression of the T-cell receptor (TCR, 5.8-fold), responsible for the primary costimulatory signal for T-cell activation, CD86 (1.9-fold) that provides a secondary positive signal, as well as the binding partner of CD86, T-cell inhibitory CTLA4 (2-fold) [41], suggested the need to tightly regulate T-cell-mediated processes. IL-18-dependent polarisation of Th responses towards the Th1 and Th17 lineages is consistent with the increased level of IL-22 observed in the chronically inflamed intestine [42]. Preferential expression of pro-inflammatory Th responses can promote a wide range of pathological responses in the intestine, mediated either by T-cells or by excessive innate immune activation [43].

Activation of TNFalpha dependent responses including induction of NFkB and the respiratory burst are typical for myeloid cells (neutrophils recruited from circulation and / or resident macrophages). This was further supported by the substantial remodelling of ECM, as evidenced by a number of significantly affected genes encoding proteins involved in ECM deposition and degradation. The high destructive power of the effectors explains severe tissue damages. In the healthy intestine, basal immune activation maintains barrier function and commensal microflora composition; however, excessive and uncontrolled inflammation likely represents a central contributor to the pathophysiology of the feed-induced distal enteritis in salmon. Microarray data revealed 11-fold induction of annexin-A1, which together with the induction of phospholipase D2, involved in processing of the annexin-1 receptor (formyl peptide receptor), suggested promotion of the pathway that can result in the inhibition of the transendothelial migration of neutrophils [44]. Suppression of the recruitment of leukocytes into the mucosa by reducing leukocyte-endothelial adhesive interactions could be an attempt to abrogate exaggerated immune responses. However, annexins may also inhibit biosynthesis of eicosanoids and therefore potentially reduce production of prostaglandin E2 (PGE2) that has a crucial role in multiple gastrointestinal defences [45]. The context of immune activation is crucial; although immune effector cells play essential roles in protective immunity against harmful luminal agents, similar effector functions seemed to be engaged during inappropriate inflammatory responses against dietary antigens. Although previous work from our group did not find a protective effect of oxytetracycline against enteritis in salmon [39], the need to engage in handling of commensal flora that breached damaged mucosal barrier should not be ruled out. Further investigation of the involvement of other, oxytetracycline resistant bacteria in the soy induced inflammatory response is warranted.

Activation of phagocytes such as neutrophils and macrophages was further indicated by the regulation of several components of the respiratory burst complex. These findings are important because neutralization of ROS represents an important defence against self-inflicted damage. Decreased mRNA levels of several glutathione-s-transferases and the key antioxidant enzymes catalase and glutathione peroxidase indicated that animals could be vulnerable to oxidative stress. Similar observations have been made in salmon and rainbow trout hepatic transcriptome after restricted feeding, SBM feeding and infection [22,46,47]. The coordinated decrease of genes encoding iron and heme proteins may have also influenced redox status. Excessive amounts of toxic metals may be prevented from entering the body by retention in the gut tissue bound to specific proteins such as metallothionein (MT) and ferritin [48]. Decreased MT and ferritin levels may thus have resulted in increased susceptibility to metal toxicity. Interaction between the regulation of inflammation and biotransformation of toxic compounds has been observed under various conditions [49,50]. Up-regulation of the NFkB pathway may be necessary to activate protection against cellular stress, indicated by the expression pattern of several heat shock proteins and ubiquitin (up 10-fold). However, NFkB suppresses AhR, which co-ordinates transcription of genes involved in xenobiotic metabolism [51]. The down-regulation of biodegradation in the intestine observed in this study may possibly increase vulnerability of the intestinal tissue and consequent hepatic loading.

The mucosal epithelial barrier of the alimentary tract is continuously exposed to noxious and immunogenic substances, including pathogens, dietary antigens and toxins. Decreased numbers of goblet cells, reduced mucus secretion and abnormalities of its composition are well described in a number of intestinal disorders [52-54]. In the present work, increased mucus production, as indicated by increased number of goblet cells, likely increased barrier properties and provided a degree of protection. In accordance, microarray data suggested augmented production of mucin glycoproteins. Notably AGA, which targets mucin glycoproteins for degradation, was down-regulated while B3GN7, which modifies glycoproteins, was 4-fold up-regulated. Another crucial aspect of the mucosal barrier is cell-cell adherence. Paracellular passage of luminal content is restricted by TJs that seal the most apical space between intestinal epithelial cells. Knudsen and co-workers suggested that soyasaponins might increase transepithelial inflow of dietary antigens and microflora through increased TJ permeability in salmon distal intestine [16]. Although molecular components of the TJ complex and their individual contributions to barrier function within the intestinal epithelium of Atlantic salmon have not been studied extensively, up-regulation of the major protein of mammalian TJs occludin could be interpreted as a sign of extensive junctional reorganization during assembly of new junctions and/or an attempt to increase TJ strength. Multiple TJ components interact with the actin cytoskeleton through binding to PDZ domains found on cytoplasmic adapter proteins, a number of which was induced in the current work. Furthermore, activation of actinomyosin contractility that leads to increased paracellular permeability was suggested by up-regulation of several myosins and RhoGTPase signaling (RhoG and RhoGEF) and suppression of the inhibitory myosin phosphatase-Rho interacting protein (see [55] for review on factors involved in regulation of TJ functions). The microarray data set was enriched for genes involved in arginine and proline metabolism (Table 1). Polyamines produced from arginine have previously been shown to be essential both in early mucosal restitution by cell migration and in regeneration by proliferation [56]. Intracellular polyamine levels are tightly regulated by the activity of ODC and ARG, which were both induced by saponins. In addition to being responsible for the generation of polyamines, ODC and ARG have been shown to be protective in a mouse model of colitis by competitive inhibition of NO production [57].

Collectively, gene expression data support the proposal that continuous cell renewal and an increased need to replace lost cells may come at the expense of proper differentiation of intestinal cells. This could partly explain the observed profile of genes involved in digestive processes and is in line with previously made observations that SBM-induced enteritis affects differentiation of epithelial cells in salmon [39]. The general down-regulation of digestive proteases was consistent with our study on SBM inclusion in salmon diets [22] and the reduced brush border enzyme activities observed in salmon fed plant-based diets such as SBM and PPC [13,14]. Dys-regulation of proteolytic actions has also been described for inflammatory bowel diseases (IBD), which shows similarities to fish enteropathy [58]. Pancreatic secretory trypsin inhibitors were found to be markedly reduced in the colon of patients with IBD [59], whereas MMP levels were elevated [60]. In the present study, decreased faecal dry matter in fish fed the PPC + S diet suggested that these fish had diarrhea [18], similar to what has been reported in salmon with SBM-induced enteritis [12]. In accordance, microarray data indicated disruption of water and solute absorption. The observed down-regulation of aquaporins is in accordance with several mammalian IBD studies [61-63], and the role of Aqp8 (down 14-fold) as a key water channel in the intestinal tract of salmonids [64]. Another interesting finding was increased levels of guanylin, which may decrease intestinal fluid absorption, increase chloride secretion and cause diarrhea in mammalian models [65]. It is known that SBM causes increased permeability of the distal intestinal epithelium [66], which likely disrupts water and ion balance. Furthermore, the observed decreased expression of nutrient transporters in the current work is in line with decreased carrier-mediated transport after SBM feeding [66].

As presented elsewhere [18], apparent digestibility of cysteine decreased when saponins were added to the PPC containing diet, indicating reduction in sulfur containing amino acids. Cysteine is a precursor for taurine, and decreased cysteine uptake together with a marked effect on cysteine deoxygenase 1 (CDO1, 46-fold down-regulated) as well as down-regulation of taurine transporter mRNA levels may have consequences for taurine biosynthesis and subsequently conjugated bile salt levels. Additionally, reduced cysteine uptake may negatively affect synthesis of the highly cysteine-rich MTs [48]. The observed transcriptional effects on MT and taurine metabolism as well as the reduced bile salt levels may therefore have resulted from reduced cysteine uptake when saponins were added to the PPC diet. In general, SBM inclusion in fish feed has been associated with decreased lipid digestibility, reduced bile salt levels and hypocholesterolemia [13,67-74]. In the present study, similar negative effects of saponins on lipid and fatty acid digestibility were observed (data presented elsewhere [18]), and microarray analyses revealed dramatic suppression of lipid and steroid metabolism. This could result both from activation of immunity and limited absorption of nutrients as a consequence of dys-regulated cellular differentiation and loss of function, as previously mentioned. Our previous studies with the 1.8 k cDNA microarray revealed similar changes in the liver of salmon infected with ISA virus [75] and the same tendency was caused with restricted feeding in salmon and rainbow trout [22,47] and by SBM inclusion in feed for salmon [22,76] and halibut [23]. It remains unknown whether suppression of lipid metabolism in the intestine could affect the condition of fish or biosynthesis of steroid hormones that require cholesterol. However, down-regulation of cholesterol and sulfate metabolism likely impaired production of bile. In accordance, saponins reduced bile salt concentration in fish fed PPC by 60% in the pyloric intestine and 56% in the mid intestine [18]. Given the key role of bile in lipid digestion and absorption, this could partly explain the observed decrease in lipid digestibility. Additionally, bile salts have several signaling properties regulating metabolic, detoxifying, antibacterial and immunomodulatory actions [77], which may have been compromised and could account for some of the observed transcriptional responses.

## Conclusions

This study promoted development of a model of feed induced intestinal inflammation in salmon. Multiple gene expression profiling further characterized the inflammation and described the intestinal pathology at the molecular level. A number of potential diagnostic markers were found, including lectin-like proteins with unknown functions in fish, aquaporins and several enzymes involved in lipid, amino acid and xenobiotic metabolism. In addition, activation of multiple mucosal defence mechanisms was outlined.

## Methods

### Diets

Five plant protein sources, corn gluten (CG; *Zea mays* L.), pea protein concentrate (PPC; *Lathyrus aphaca*), sunflower meal (SFM; *Helianthus anuus*), rapeseed meal (RSM; *Brassica napus*) and horsebean meal (HBM; *Vicia faba* var. *equina*) were investigated without and with 2 g kg^−1^ soyasaponin supplementation (± S, ten diets in total). The saponin supplementation level corresponded to a level provided by a 40% SBM dietary inclusion. Formulation of the experimental diets is presented in Table 6. The diets were formulated to contain a crude protein (CP) to energy ratio of 20 g MJ^−1^. As the fibre and protein content of the selected plant sources differed greatly, two levels of dietary protein replacement were used; CG and PPC were included at a level corresponding to 33% of total protein, while SFM, RSM and HBM were at 21%. Dietary energy level was allowed to vary to avoid using fillers in the diets for adjusting the energy level, as fillers often have side effects that may influence results. In all of the diets, protein from the various plant sources partially replaced marine fish protein derived from a combination of Nordic LT and South American Superprime fishmeals. All diets were supplemented with standard vitamin and micromineral premixes and contained 100 mg kg^−1^ yttrium oxide as an inert marker for calculation of nutrient apparent digestibilities. The diets were produced by extrusion at the BioMar Feed Technology Centre (Brande, Denmark) with a pellet size of 5 mm in batches of 50 kg. Chemical composition of the diets is presented in detail elsewhere [18].

**Table 6:** Formulation of the diets (%)

**Ingredients**	**CG + S**	**PPC + S**	**SFM + S**	**RSM + S**	**HBM + S**
Nordic LT-meal	22.3	20.7	25.3	23.9	23.4
Superprime Fish meal	22.3	20.7	25.3	23.9	23.4
Corn gluten	25.2				
Pea protein concentrate		30.2			
HP sunflower			22.1		
Rapeseed meal				26.3	
Horse beans					33.5
Saponins	0.2	0.2	0.2	0.2	0.2
Tapioka	6.0	6.0	6.0	6.0	0.0
Fish oil	11.8	10.7	10.4	9.9	9.6
Rapeseed oil	11.8	10.7	10.4	9.9	9.6
Vitamin-Mineral Mix	0.38	0.38	0.38	0.38	0.38
Lysine	0.21				
DL-Methionine		0.37			
Carophyll Pink	0.04	0.04	0.04	0.04	0.04
Monocalcium phosphate	0.30	0.51			

*CG* corn gluten, *PPC* pea protein concentrate, *SFM* sunflower meal, *RSM* rapeseed meal and *HBM* horse bean meal supplemented with a 95% soyasaponin extract (+S) at the rate of 2 g kg^−1^ diet.

The respective control diets for each plant protein source were identical except for S inclusion.

### Experimental animals, conditions and sampling

The present experiment was approved by the Norwegian Animal Research Authority and conducted according to prevailing animal welfare regulations. The feeding trial was performed at Nofima Marin research station at Sunndalsøra, Norway. Atlantic salmon (*Salmo salar* L.) post smolts of the Sunndalsøra breed with mean weight of 270 g ± 10% were allocated in fiberglass tanks (1 m^3^, 30 fish tank^−1^) with flow-through seawater (500 L, flow rate 20 L min^−1^). Two replicate tanks per diet (20 tanks in total) were used. Water temperature varied between 9 and 13°C. Oxygen content and salinity of the outlet water were monitored to secure saturation above 85% and stability, respectively. A 24 h lighting regime was employed during the experimental period. The fish were fed to satiation using automatic disc feeders giving out feed every 10 min and which were refilled every 3 days. The feeding trial ran for 80 days. Tank sampling order and fish sampling were conducted randomly. Twelve fish were sampled from each tank and euthanized by overdosing with tricaine methane-sulfonate (MS-222). All sampled fish had the peritoneal cavity opened and the gastrointestinal tract taken out and cleaned free of adipose tissue. To ensure intestinal exposure to the diets, only fish with digesta throughout the intestinal tracts were sampled. Approximately 300 mg of the distal intestinal (DI) segments were placed in RNAlater (Ambion®, Life Technologies, Carlsbad, CA, USA) at 4°C for 24 h and then stored at −20°C. Histology samples were taken from the DI, fixed in phosphate-buffered formalin (4% formaldehyde) for 24 h and then transferred to 70% ethanol until processing.

### RNA extraction

Total RNA was extracted from DI tissue samples (~50 mg) using Trizol® reagent (Invitrogen™, Life Technologies, Carlsbad, CA, USA) and purified with Pure Link (Invitrogen™) including an on-column DNase treatment according to the manufacturer’s protocol. The integrity of the RNA samples was verified by the 2100 Bioanalyzer in combination with an RNA Nano Chip (Agilent Technologies Santa Clara, CA, USA), and RNA purity and concentrations were measured using the NanoDrop ND-1000 Spectrophotometer (Thermo Fisher Scientific, Waltham, MA, USA). Total RNA was stored at −80°C until use.

### Microarrays

Five series of microarray analyses were performed according to the number of diets. In each, four individual samples of fish (two from each tank duplicate) that received a saponin supplemented feed were compared with a pooled sample (an equalized mixture of 12 individuals, 6 from each tank duplicate) from the respective control diet without saponins. This made it possible to differentiate the effects of saponins from those caused by plant ingredients. Nofima’s Atlantic salmon oligonucleotide microarray and bioinformatic system (STARS) were used [78]. The platform includes 21 k unique probes spotted in duplicate; the genes were annotated by functions (GO), pathways (KEGG) and custom vocabulary. Microarrays were manufactured by Agilent Technologies (Santa Clara, CA, USA) and unless indicated otherwise, the reagents and equipment were from the same source. RNA amplification and labeling were performed with a Two-Colour Quick Amp Labelling Kit and a Gene Expression Hybridization kit was used for fragmentation of labeled RNA. Target samples were labeled with Cy5 and Cy3 was used for controls. The input of total RNA used in each reaction was 500 ng. After overnight hybridization in an oven (17 h, 65°C, rotation speed 10 rpm), arrays were washed with Gene Expression Wash Buffers 1 and 2 and scanned with a GenePix 4100A (Molecular Devices, Sunnyvale, CA, USA). GenePix Pro 6.0 was used for spot to grid alignment, assessment of spot quality, feature extraction and quantification. Subsequent data analyses were performed with STARS. After filtration of low quality spots flagged by GenePix, Lowess normalization of log_2_-expression ratios (ER) was performed. The differentially expressed genes (DEG) were selected by difference from control and expression change (one sample t test, p < 0.05 and >1.6-fold). Hierarchical clustering of samples was performed by Euclidian distances using Wards’ method for construction of a tree; 993 genes affected by saponins in at least one study group were included in the analysis. Enrichment of GO and KEGG terms in the list of DEG was assessed with Yates’ corrected chi-square using all probes that passed quality control as a reference; enriched terms corresponding to at least five differentially expressed genes were selected. Complete data files were deposited in NCBI’s Gene Expression Omnibus with accession number GSE34578.

### Quantitative real time PCR (qPCR)

For validation of microarray results with qPCR, fifteen genes were selected that represented the major functional classes affected by the treatment (Table 2). qPCR was performed according to MIQE standards [79] on 9 animals from each diet group (4–5 individuals from each tank duplicate). First strand cDNA synthesis was performed using 1.0 μg total RNA from all samples using Superscript III (Invitrogen™, Life Technologies, Carlsbad, CA, USA) in 20 μL reactions, and primed with Oligo(dT)_20_ primers according to the manufacturer's protocol. Negative controls were performed in parallel by omitting RNA or enzyme. Obtained cDNA was diluted 1:10 before use and stored at −20°C. qPCR primers were designed using Primer3 software (http://frodo.wi.mit.edu/primer3/). Primer details are shown in Table 2. All primer pairs gave a single band pattern for the expected amplicon of interest in all reactions. PCR reaction efficiency (E) for each gene assay was determined using 10-fold serial dilutions of randomly pooled cDNA. Expression of individual gene targets was analyzed using the LightCycler 480 (Roche Diagnostics). Each 10 μL DNA amplification reaction contained 2 μL PCR-grade water, 2 μL of 1:10 diluted cDNA template, 5 μL of Lightcycler 480 SYBR Green I Master (Roche Diagnostics, Basel, Switzerland) and 0.5 μL (final concentration 500 nM) of each forward and reverse primer. Each sample was assayed in duplicate, including a no template control (NTC). The three-step qPCR program included an enzyme activation step at 95°C (5 min) and 40 cycles of 95°C (10 s), 60°C (10 s) and 72°C (15 s). Quantification cycle (C_q_) values were calculated using the "second derivative maximum method" measuring maximum increase rate of newly synthesized DNA per cycle was used on the basis of the LightCycler 480 software release 1.5.0 (Roche Diagnostics, Basel, Switzerland). To confirm amplification specificity the PCR products from each primer pair were subjected to melting curve analysis and visual inspection of PCR products after each run by agarose gel electrophoresis. EF1A, HPRT1, GAPDH and RNAPOLII were evaluated for use as reference genes (Table 2) by ranking relative gene expression according to their overall coefficient of variation (CV) and their interspecific variance as described previously [80]. GAPDH was used as a normalization factor, as it showed stable expression pattern between individuals, and no significant differences were observed between dietary groups. Relative expression of target genes was evaluated using the ΔΔC_T_ method [81]. Significant differences were assessed using Student’s T-test with a significance level of p < 0.05.

### Histology

Histology samples were processed using standard histological techniques and stained with haematoxylin and eosin (H&E) at the Norwegian School of Veterinary Science. Distal intestine tissue was sectioned in a longitudinal plane. Tissue sections were evaluated by light microscopy in randomized order.

## Competing interests

The authors declare that they have no competing interests.

## Authors’ Contributions

TMK, SS and AK drafted the manuscript and performed gene expression analyses. MHP performed histopathological analysis. LTM and BD participated in gene expression analyses. MH and ÅK designed and conducted the feeding trial. All authors have read and approved the final manuscript.
